# Editorial: Assembly of the Photosystem II Membrane-Protein Complex of Oxygenic Photosynthesis

**DOI:** 10.3389/fpls.2017.00884

**Published:** 2017-05-26

**Authors:** Julian J. Eaton-Rye, Roman Sobotka

**Affiliations:** ^1^Department of Biochemistry, University of OtagoDunedin, New Zealand; ^2^Laboratory of Photosynthesis, Center Algatech, Institute of Microbiology, Czech Academy of SciencesTřeboň, Czechia; ^3^Faculty of Science, University of South BohemiaČeské Budějovice, Czechia

**Keywords:** *Arabidopsis thaliana*, cyanobacteria, *Chlamydomonas reinhardtii*, biogenesis, photodamage, Photosystem II, photosynthesis, *Synechocystis* sp. PCC 6803

Photosystem II (PS II) catalyses the light-driven splitting of water at the start of the photosynthetic electron transport chain found in the thylakoid membranes of plants, algae and cyanobacteria (Barber, 2016; Vinyard and Brudvig, 2017). The mature photosystem is dimeric and able to form super-complexes with a range of pigment-binding antenna proteins found across the different phyla (Shen, 2015; Ago et al., 2016; Wei et al., 2016). The highest resolution X-ray-derived crystal structure of PS II is from the thermophilic cyanobacterium *Thermosynechococcus vulcanus* where each monomer is composed of 20 protein subunits and more than 80 cofactors as well as over a 1,000 bound water molecules (Umena et al., 2011; Suga et al., 2015). The precise arrangement of redox co-factors, pigments and other organic and inorganic co-factors is the product of multiple ordered processes requiring spatial and temporal coordination and the participation of numerous protein assembly factors (Nickelsen and Rengstl, 2013).

In this Frontiers Research Topic 25 articles have been collected under six different sections addressing different aspects of PS II assembly. The first section contains three contributions covering the **Evolutionary origins and photoactivation** of PS II. The first of these by Cardona considers the origin of the PS II reaction center D1 and D2 proteins that bind the majority of the PS II redox active cofactors, he also considers afresh the origin of the two chlorophyll-binding proximal antenna proteins, CP43 and CP47 as well as the origins of the additional membrane-spanning low-molecular-weight subunits of the photosystem. Finally this first article considers the events leading to the origin of the Mn_4_CaO_5_ cluster of the oxygen-evolving complex that catalyzes water splitting. An in-depth treatment of the assembly of the Mn_4_CaO_5_ cluster then follows in the review by Bao and Burnap and the final article in this section by Ifuku and Noguchi discusses the roles of the lumenal PS II subunits that provide a proteinaceous cap for the oxygen-evolving complex.

Each PS II monomer contains 35 chlorophylls, two pheophytins, 11 β-carotenes as well as over 20 lipids (Umena et al., 2011). So far, however, it is not clear how the synthesis of potentially phototoxic chlorophyll molecules is coordinated with the synthesis of chlorophyll-binding PS II subunits. A mechanistic explanation of pigment insertion into PS II proteins is also not available. Section 2 of the topic, **Pigment biosynthesis and lipids**, includes examples of how chlorophyll synthesis and the presence of other pigments and lipids are integral to the ordered assembly of PS II centers. The report by Hollingshead et al. establishes that restricting *de novo* chlorophyll biosynthesis, in this case by modulating Mg-protoporphyrin IX methyl ester cyclase activity, affects synthesis of the PS II subunits CP43 and CP47 along with Photosystem I (PS I) but no other chlorophyll-containing proteins, including D1 and D2. A similar alteration in chlorophyll production was achieved by Crawford et al. who introduced a targeted mutation into Mg-chelatase, the enzyme responsible for the first-committed step of chlorophyll biosynthesis. Crawford et al. found that restricting chlorophyll availability in a cyanobacterial strain lacking the Ycf48 assembly factor increased the severity of removing this protein. The results of Hollingshead et al. and Crawford and colleagues indicate a different mode of chlorophyll loading into the D1 and D2 subunits than into other chlorophyll-binding proteins and suggest a role for assembly factors in chlorophyll insertion or turnover. Also in section 2, Zakar et al. review the different roles of carotenes and xanthophylls in cyanobacterial PS II assembly and function and finally the report by Kobayashi et al. concludes this section by showing that plastidic phosphatidylglycerol is required for PS II function in *Arabidopsis thaliana*. It is known that the absence of this lipid in cyanobacterial thylakoids affects electron transfer between Q_A_ and Q_B_, the primary and secondary quinone electron acceptors of PS II, respectively (Gombos et al., 2002). Interestingly, Kobayashi et al. demonstrate that the absence of phosphatidylglycerol in *A. thaliana* also disrupts energy transfer from the antenna to PS II and increases the susceptibility of PS II to photodamage induced by red light.

To date over 30 polypeptides have been identified as PS II assembly factors that participate in biogenesis but are not present in the mature complex. These include kinases, phosphatases, transporters and proteases in addition to those acting as chaperones (Järvi et al., 2015). Currently, an explanation of the exact function of any of these factors in the PSII-assembling machinery is missing and undoubtedly many more factors remain to be discovered (Nickelsen and Rengstl, 2013). In the section **Photosystem II assembly factors**, the review by Lu considers the molecular functions of the many proteins that influence PS II assembly and its repair following photodamage in *A. thaliana*. The next article by Plochinger et al. focuses on the PsbW, PsbY, HCF136, PsbN, TerC, and ALB3 proteins, although among these proteins, PsbY is, in fact, present in the final holoenzyme (Shen, 2015). How thylakoid membrane biogenesis and PS II assembly processes are coordinated is also an area of keen interest (Nickelsen et al., 2011). The report by Rast et al. addresses this topic by investigating the function of the Slr0151 tetratricopeptide repeat protein on thylakoid ultrastructure during PS II assembly and repair in the cyanobacterium *Synechocystis* sp. PCC 6803. Adding to the complexity of PS II assembly are examples where assembly factors appear to have more than one role. The thylakoid lumenal protein TLP18.3 (Psb32 in cyanobacteria) has been shown to influence the repair cycle (Sirpiö et al., 2007) but when Järvi et al. investigated the function of TLP18.3 during fluctuating light in *A. thaliana*, the absence of this protein disrupted expression of genes involved in plant defense processes such that *tlp18.3* plants exhibited stunted growth. The Psb27 protein may also have a dual role influencing biogenesis of both PS I and PS II, although its major contribution appears to be on PS II assembly (Komenda et al., 2012a). The report by Cormann et al. presents new data supporting an interaction of Psb27 with CP43 [as reported by Komenda et al. (2012a) and Liu et al. (2011)] as well as interactions with CP47 and the C-termini of both D1 and D2. The results of Cormann et al. suggest a binding site for Psb27 that is occupied by the lumenal PsbV protein in the mature complex of cyanobacterial PS II.

The existence of two distinct assembly pathways: *de novo* biogenesis and the specialized cycle to repair photodamaged photosystems, is a unique property of PS II (Mulo et al., 2012). Photodamage is an inevitable consequence of the oxidative chemistry of water splitting and is chiefly caused by reactive oxygen species generated both by electron transfer and energy transfer processes (Vass, 2012). The location of these pathways within the thylakoid membrane system and extent to which they may share intermediate complexes, as well as auxiliary assembly factors, remains to be established but may in fact differ between cyanobacteria and algae and potentially between lower and higher plants (Komenda et al., 2012b). Section 4 contains several papers dealing with the **Assembly and maintenance of Photosystem II**. Weisz et al. review recent developments in mass spectrometry that are increasingly contributing to the characterization of the individual PS II sub-complexes that participate in assembly and turnover during biogenesis, photodamage and repair. Michoux et al. analyze the impact of truncating the N-terminal tail of the D1 protein in tobacco. This truncation led to the loss of PS II super-complexes and dimeric complexes in the thylakoid membrane but unlike *Synechocystis* sp. PCC 6803, where the N-terminus of D1 has been shown to be involved in the degradation of photodamaged PS II by FtsH complexes (Komenda et al., 2007), the data from Michoux et al. indicate that tobacco has additional compensatory pathways for regulating D1 turnover when the N-terminus of D1 is removed. It is also known that the Deg1 protease is involved in the repair cycle in *A. thaliana* [for a mechanism see (Kley et al., 2011)] and the paper by Cheregi et al. provides a perspective on the roles of Deg/HtrA proteases in cyanobacteria. A number of assembly factors participate in assembly steps directly or in concert with transcriptional or translational control. In eukaryotes, nuclear factors are required in the regulation of gene expression for plastid-encoded PS II proteins. Working with *Chlamydomonas reinhardtii*, Munir et al. report the role of the nuclear gene *TBC1* in regulating the plastid *psbC* gene that encodes CP43.

Absorption of photons by intermediate pre-assembly complexes is likely to impair biogenesis via the production of reactive oxygen species. This is an important topic that has not yet received the full attention it deserves. It has long been recognized, however, that cytochrome *b*_559_, an essential component of the PS II reaction center, may function in a protective side branch pathway for electron transport (Rutherford et al., 2012). The last contribution in this section is a mini review by Chu and Chiu focused on the roles of this cytochrome in PS II assembly and photoprotection.

A broad spectrum of environmental factors influence biogenesis and turnover of PS II and four examples are presented in section 5, **Environmental influences**. In the first contribution, Schoffman et al. review the impact of iron on the availability of several macro and micro nutrients utilized in energy transduction and biochemical catalysis in phytoplankton. On a different tack, both chloroplasts and mitochondria retain genomes encoding certain proteins belonging to bioenergetic protein complexes (Allen and Martin, 2016). The chloroplast sensor kinase CSK in *A. thaliana* has been shown to regulate plastid gene expression in response to the redox status of the electron transport chain (Puthiyaveetil et al., 2008). In the report by Ibrahim et al. the cyanobacterial homolog of CSK is shown to exhibit redox dependent, and Na^+^ ion dependent, phosphoryl group transfer to two response regulators and this pathway is proposed to control photosystem stoichiometry. Environmental factors other than light may impact on PS II performance by influencing the thylakoid lumen. It has been observed that several cyanobacterial mutants lacking different combinations of extrinsic PS II proteins are not photoautotrophic at pH 7.5 and their PS II assembly is impaired; however, the cells are photoautotrophic at pH 10 (Summerfield et al., 2013). The perspective provided by Morris et al. discusses possible mechanisms for coupling the environmental pH to the regulation of PS II assembly and activity in mutants lacking specific PS II lumenal proteins. Finally in this section, Bielczynski et al. report the effect of high light on antenna organization and show that acclimation to high light in *A. thaliana* results in a reduction in functional antenna size that exceeds the actual reduction of antenna proteins. In addition, the authors observed an increase in light-harvesting complex II (LHCII) monomers in plants acclimated to high light but they did not observe a corresponding change in the LHCII trimer to monomer ratio during short exposures to light stress.

The final section of this Research Topic has been reserved for papers offering **Methodological and technical considerations**. In the opening opinion piece of this section Rühle and Leister argue for a synthetic biological approach to building PS II using a cyanobacterial chassis that could identify the minimal suite of proteins to assemble a functional photosystem. The research report by Tichý et al. highlights the genome flexibility of *Synechocystis* sp. PCC 6803 during laboratory cultivation and demonstrates that even large chromosomal rearrangements are possible. This study shows that it is essential to establish that the correct control strain is being compared to when analyzing cyanobacterial mutants. In the perspective from Rehman et al. the authors demonstrate that the antibioitic chloramphenicol can accept electrons from PS II and transfer them to oxygen giving rise to superoxide production. Chloramphenicol has been widely used in photodamage studies to separate effects on the rate of damage from effects on recovery due to protein synthesis. This study therefore suggests that caution must be exercised when interpreting photodamage studies in the presence of chloramphenicol. The final contribution by Haniewicz et al. presents a protocol for isolating different PS II populations in thylakoid membranes from tobacco and emphasizes the ability to obtain high yields of PS II-LHCII super-complexes.

## Concluding comment

Oxygenic photosynthesis first evolved ~3 billion years ago resulting in the transition from an anaerobic to an aerobic atmosphere (Lyons et al., 2014). This led to the protective ozone layer and the advent of aerobic respiration that paved the way for the formation and success of the eukaryotic cell (Martin et al., 2015). The chemistry of PS II is therefore responsible for almost all of our planet's biodiversity; however, as noted above, this fundamental process comes with a cost. The oxidative chemistry of water splitting inescapably produces reactive oxygen species and radicals that damage PS II and require the photosynthetic machinery to be continually renewed (Vass, 2012; Nishiyama and Murata, 2014). As the examples in this Research Topic show, in addition to *de novo* biogenesis, PS II possesses a self-healing cycle leading to the rate of repair keeping pace with the rate of light-induced photodamage. Environmental conditions, such as extreme temperatures or excessive light levels, can tip the balance such that repair cannot keep up with damage leading to reduced photosynthetic yields (Murata et al., 2007). A deeper understanding of how plants repair PS II to prolong the lifetime of the enzyme will provide new approaches to the design of hardier crop plants. Alongside this, studies of PS II biogenesis will deepen our understanding of how the catalytic oxygen-evolving center is assembled and provide novel insight into the origin and evolution of oxygenic photosynthesis. These avenues of research will also inform the design of biomimetic systems for the production of hydrogen fuel and electrons from water (Blankenship et al., 2011; Najafpour et al., 2016). Current projections of population growth indicate we will reach 8.5 billion by 2,030 and exceed 11 billion by 2,100 (United Nations, 2015). Research into the assembly of PS II will directly contribute to our food and energy security and benefit these future generations.

## Author contributions

JE initiated this research topic. For the editorial both authors reviewed all Research Topic articles. JE wrote the first draft and JE and RS revised and prepared the final version. Both authors approved it for publication.

### Conflict of interest statement

The authors declare that the research was conducted in the absence of any commercial or financial relationships that could be construed as a potential conflict of interest.

## References

[B1] Ago H. Adachi H. Umena Y. Tashiro T. Kawakami K. Kamiya N.. (2016). Novel features of eukaryotic Photosystem II revealed by its crystal structure analysis from a red alga. J. Biol. Chem. 291, 5676–5687. 10.1074/jbc.M115.711689 26757821 PMC4786707

[B2] Allen J. F. Martin W. F. (2016). Why have organelles retained genomes? Cell Systems 2, 71–72. 10.1016/j.cels.2016.02.007 27135161

[B3] Barber J. (2016). Photosystem II: the water splitting enzyme of photosynthesis and the origin of oxygen in our atmosphere. Q. Rev. Biophys. 49:e14 10.1017/s0033583516000123 27659174

[B4] Blankenship R. E. Tiede D. M. Barber J. Brudvig G. W. Fleming G. Ghirardi M.. (2011). Comparing photosynthetic and photovoltaic efficiencies and recognizing the potential for improvement. Science 332, 805–808. 10.1126/science.1200165 21566184

[B5] Gombos Z. Varkonyi V. Hagio M. Iwaki M. Kovács L. Masamoto K. (2002). Phosphatidylglycerol requirement for the function of electron acceptor QB in the Photosystem II reaction center. Biochemistry 41, 3796–3802. 10.1021/bi011884h 11888298

[B6] Järvi S. Suorsa M. Aro E.-M. (2015). Photosystem II repair in plant chloroplasts—regulation, assisting proteins and shared components with Photosystem II biogenesis. Biochim. Biophys. Acta 1847, 900–909. 10.1016/j.bbabio.2015.01.006 25615587

[B7] Kley J. Schmidt B. Boyanov B. Stolt-Bergner P. C. Kirk R. Ehrmann M.. (2011). Structural adaptation of the plant protease Deg1 to repair Photosystem II during light exposure. Nat. Struct. Mol. Biol. 18, 728–731. 10.1038/nsmb.2055 21532594

[B8] Komenda J. Knoppová J. Kopečná J. Sobotka R. Halada P. Yu J.. (2012a). The Psb27 assembly factor binds to the CP43 complex of Photosystem II in the cyanobacterium Synechocystis sp. PCC 6803. Plant Physiol. 158, 476–486. 10.1104/pp.111.184184 22086423 PMC3252115

[B9] Komenda J. Sobotka R. Nixon P. J. (2012b). Assembling and maintaining the Photosystem II complex in chloroplasts and cyanobacteria. Curr. Opin. Plant Biol. 15, 245–251. 10.1016/j.pbi.2012.01.017 22386092

[B10] Komenda J. Tichy M. Prášil O. Knoppová J. Kuviková S. de Vries R.. (2007). The exposed N-terminal tail of the D1 subunit is required for rapid D1 degradation during Photosystem II repair in Synechocystis sp. PCC 6803. Plant Cell 19, 2839–2854. 10.1105/tpc.107.053868 17905897 PMC2048700

[B11] Liu H. Huang R. Y.-C. Chen J. Gross M. L. Pakrasi H. B. (2011). Psb27, a transiently associated protein, binds to the chlorophyll binding protein CP43 in Photosystem II assembly intermediates. Proc. Natl. Acad. Sci. U.S.A. 108, 18536–18541. 10.1073/pnas.1111597108 22031695 PMC3215006

[B12] Lyons T. W. Reinhard C. T. Planavsky N. J. (2014). The rise of oxygen in Earth's early ocean and atmosphere. Nature 506, 307–315. 10.1038/nature13068 24553238

[B13] Martin W. F. Garg S. Zimorski V. (2015). Endosymbiotic theories for eukaryotic origin. Phil. Trans. R. Soc. B 370:20140330 10.1098/rstb.2014.0330 26323761 PMC4571569

[B14] Mulo P. Sakurai I. Aro E.-M. (2012). Strategies for psbA gene expression in cyanobacteria, green algae and higher plants: from transcription to PSII repair. Biochim. Biophys. Acta 1817, 247–257. 10.1016/j.bbabio.2011.04.011 21565160

[B15] Murata N. Takahashi S. Nishiyama Y. Allakhversiev S. I. (2007). Photoinhibition of Photosystem II under environmental stress. Biochim. Biophys. Acta 1767, 414–421. 10.1016/j.bbabio.2006.11.019 17207454

[B16] Najafpour M. M. Renger G. Holyńska M. Moghaddam A. N. Aro E.-M. Carpentier R.. (2016). Manganese compounds as water-oxidizing catalysts: From the natural water-oxidizing complex to nanosized manganese oxide structures. Chem. Rev. 116, 2886–2936. 10.1021/acs.chemrev.5b00340 26812090

[B17] Nickelsen J. Rengstl B. (2013). Photosystem II assembly: from cyanobacteria to plants. Annu. Rev. Plant Biol. 64, 609–635. 10.1146/annurev-arplant-050312-120124 23451783

[B18] Nickelsen J. Rengstl B. Stengel A. Schottkowski M. Soll J. Ankele E. (2011). Biogenesis of the cyanobacterial thylakoid membrane system—an update. FEMS Microbiol. Lett. 315, 1–5. 10.1111/j.1574-6968.2010.02096.x 20831593

[B19] Nishiyama Y. Murata N. (2014). Revised scheme for the mechanism of photoinhibition and its application to enhance the abiotic stress tolerance of the photosynthetic machinery. Appl. Microbiol. Biotechnol. 98, 8777–8796. 10.1007/s00253-014-6020-0 25139449

[B20] Puthiyaveetil S. Kavanagh T. A. Cain P. Sullivan J. A. Newell C. A. Gray J. C.. (2008). The ancestral symbiont sensor kinase CSK links photosynthesis with gene expression in chloroplasts. Proc. Natl. Acad. Sci. U.S.A. 105, 10061–10066. 10.1073/pnas.0803928105 18632566 PMC2474565

[B21] Rutherford A. W. Osyczka A. Rappaport F. (2012). Back-reactions, short-circuits, leaks and other energy wasteful reactions in biological electron transfer: Redox tuning to survive life in O2. FEBS Lett. 586, 603–616. 10.1016/j.febslet.2011.12.039 22251618

[B22] Shen J.-R. (2015). The structure of Photosystem, II, and the mechanism of water oxidation in photosynthesis. Annu. Rev. Plant Biol. 66, 23–48. 10.1146/annurev-arplant-050312-120129 25746448

[B23] Sirpiö S. Allahverdiyeva Y. Suorsa M. Paakkarinen V. Vainonen J. Battchikova N.. (2007). TLP18.3, a novel thylakoid lumen protein regulating photosystem II repair cycle. Biochem. J. 406, 415–425. 10.1042/BJ20070460 17576201 PMC2049043

[B24] Suga M. Akita F. Hirata K. Ueno G. Murakami H. Nakajima Y.. (2015). Native structure of Photosystem II at 1.95 Å resolution viewed by femtosecond X-ray pulses. Nature 517, 99–103. 10.1038/nature13991 25470056

[B25] Summerfield T. C. Crawford T. S. Young R. D. Chua J. P. S. Macdonald R. L. Sherman L. A.. (2013). Environmental pH affects photoautotrophic growth of Synechocystis sp. PCC 6803 strains carrying mutations in the lumenal proteins of PSII. Plant Cell Physiol. 54, 859–874. 10.1093/pcp/pct036 23444302

[B26] Umena Y. Kawakami K. Shen J.-R. Kamiya N. (2011). Crystal structure of oxygen-evolving Photosystem II at a resolution of 1.9 Å. Nature 473, 55–60. 10.1038/nature09913 21499260

[B27] United Nations (2015). World Population Prospects: The 2015 Revision, Key Findings and Advance Tables. Department of Economic and Social Affairs, Population Division, Working paper No. ESA/P/WP.241, United Nations.

[B28] Vass I. (2012). Molecular mechanisms of photodamage in the photosystem II complex. Biochim. Biophys. Acta 1817, 209–217. 10.1016/j.bbabio.2011.04.014 21565163

[B29] Vinyard D. J. Brudvig G. W. (2017). Progress towards a molecular mechanism of water oxidation in Photosystem, II. Annu. Rev. Phys. Chem. 68, 101–116. 10.1146/annurev-physchem-052516-044820 28226223

[B30] Wei X. Su X. Cao P. Liu X. Chang W. Li M.. (2016). Spinach Photosystem II-LHCII supercomplex at 3.2 Å resolution. Nature 534, 69–74. 10.1038/nature18020 27251276

